# Oculomotor and Caloric Test Findings in 122 Patients With Unilateral Vestibular Schwannoma

**DOI:** 10.7759/cureus.108944

**Published:** 2026-05-16

**Authors:** Suman Narayana Swamy, Pradeep Yuvaraj, Rao M Pradyumna, Nupur Pruthi, Prabhuraj AR, Kandavel Thennarasu, Ponnusamy N, Aravind Kumar Rajasekaran

**Affiliations:** 1 Speech Pathology and Audiology, National Institute of Mental Health and Neurosciences, Bangalore, IND; 2 Neurosurgery, National Institute of Mental Health and Neurosciences, Bangalore, IND; 3 Biostatistics, National Institute of Mental Health and Neurosciences, Bangalore, IND

**Keywords:** caloric test, oculomotor test, vestibular schwannoma, vestibulo-ocular reflex (vor), videonystagmography (vng)

## Abstract

Introduction

Vestibular schwannoma (VS), a benign neoplasm of the eighth cranial nerve, can disrupt both peripheral vestibular function and central oculomotor pathways. The caloric test evaluates the vestibulo-ocular reflex (VOR) pathway, while oculomotor testing assesses central neural mechanisms. In this study, caloric and oculomotor assessments were performed in patients with unilateral VS to examine peripheral and central pathway integrity.

Materials and methods

The study recruited 122 patients (aged 18-55 years) with unilateral VS. It encompassed 60 patients with right-sided VS (Rt-VS) and 62 with left-sided VS (Lt-VS). It also included 120 age- and gender-matched healthy controls. Participants underwent bi-thermal caloric and oculomotor testing, and relevant metrics were recorded. Between-group differences were assessed using the Mann-Whitney U test, and associations between tumor volume and test metrics were examined using Spearman’s correlation.

Results

The patient groups (Rt-VS and Lt-VS) revealed significant differences (p<0.05) in caloric and oculomotor tests compared to controls. In the caloric test, patient groups demonstrated significantly reduced lesion-side responses and exhibited unilateral weakness. Oculomotor assessment showed significant (p<0.05) abnormalities in saccadic, smooth pursuit, and optokinetic functions in patient groups. Saccade and optokinetic parameters were significantly affected bilaterally and in both horizontal and vertical directions. In smooth pursuit, though horizontal was reduced in both groups, vertical deficits were observed only in the Rt-VS group. Overall findings indicate central oculomotor dysfunction, along with vestibular impairment in unilateral VS.

Conclusion

The study findings confirm that VS could compromise both peripheral and central functions. Caloric test results indicate significant peripheral vestibular dysfunction, whereas oculomotor abnormalities suggest central involvement. Large tumor size could mask the benefit of the compensatory mechanisms. Together, these findings underscore the importance of combining vestibular and oculomotor assessments to achieve a more comprehensive evaluation of functional impairment in individuals with VS.

## Introduction

The vestibular system helps with gaze stabilization and postural control. It employs the vestibulo-ocular reflex (VOR) to produce compensatory eye movements matching head velocity in the opposite direction, thereby maintaining stable gaze. The VOR pathway begins at the semicircular canals and, through the vestibular nerve, the vestibular nuclei, the medial longitudinal fasciculus, and the extraocular motor nuclei, reaches the extraocular muscles [1]. In addition to the VOR, the vestibular system interacts with the central oculomotor networks. This is to maintain visual stability during motion. Smooth pursuit (continuous tracking of moving targets), saccade (rapid gaze redirection), and optokinetic eye movements (visual scene stabilization during sustained motion) constitute the coculomotor functions [2]. The VOR and oculomotor systems are mediated by distinct neural circuits. However, they converge on a common final neural pathway at the extraocular motor nuclei (III, IV, and VI), which serve as the final common output for generating eye movements

Vestibular schwannoma (VS) is a benign tumor arising from the Schwann cells of the vestibular division of the eighth cranial nerve. The earliest effects typically involve disruption of the peripheral vestibular nerve. As the tumour enlarges, compression of adjacent neural structures, including the brainstem and cerebellum, may lead to additional central deficits involving oculomotor control [3]. Disruption of peripheral vestibular input, as seen in VS, results in asymmetry between the bilateral vestibular systems. This imbalance affects both VOR and oculomotor control [4].

Videonystagmography (VNG) objectively assesses vestibular(peripheral) and central oculomotor function [5]. The VNG test battery has caloric stimulation, smooth pursuit, saccades, optokinetic responses, and gaze assessment. The caloric test utilises bithermal stimulation of the external auditory canal and is reported to be affected in patients with VS. In patients with unilateral vestibular dysfunction, asymmetrical (Right vs Left) caloric responses could be observed. Unilateral weakness (UW), directional preponderance (DP), and slow-phase velocity (SPV) are the test metrics in the caloric test. The right and left vestibular systems should give synchronous and symmetrical inputs to the higher centres. An imbalance could cause UW, indicating peripheral vestibular dysfunction. DP, conversely, indicates asymmetry in nystagmus intensity and is frequently regarded as an indicator of central vestibular imbalance. DP typically normalizes with effective compensation, while enduring abnormalities may indicate insufficient central adaptation [6-8].

VNG abnormalities have been reported in the literature [9]. Patients with VS may show varied findings on oculomotor tests. Oculomotor tests could reveal dysfunctions like reduced gain and presence of corrective (catch-up) saccades on smooth pursuit, increased latency, reduced peak velocity, and poor precision on saccades and reduced slow phase velocity in optokinetic. These abnormalities reflect central involvement caused by the direct mass effect of the VS. However, these variations may also indicate compensatory mechanisms [10]. Thus, oculomotor testing provides critical insight into central neural dysfunction and complements caloric measures of peripheral vestibular loss in patients with VS.

In cases of unilateral vestibular dysfunction, the central mechanisms try to restore functional symmetry and compensate for the peripheral vestibular dysfunction. The central compensation is a complex process and involves recalibration of neuronal activity, especially within the vestibular nuclei and the cerebellum. However, the extent of compensation varies depending on lesion severity and involvement of central structures [11]. In case central pathways are compromised, as with larger tumours affecting cerebellar or brainstem regions, both vestibular and oculomotor compensation may be limited, leading to persistent functional deficits.

Few studies have emphasised the importance of multi-test vestibular evaluation in VS, highlighting the complementary nature of different diagnostic modalities [10]. However, most studies have examined caloric or oculomotor parameters independently and often in small samples. A comprehensive peripheral and central evaluation integrating both peripheral and central measures is necessary to understand the functional impact of VS. Furthermore, the relationship between tumour characteristics, such as volume, and functional deficits remains insufficiently explored. The present study addresses this gap by using VNG to evaluate both caloric-induced vestibular responses and oculomotor function in a large cohort of patients with unilateral VS. 

## Materials and methods

This was a prospective cross-sectional study conducted at the Department of Speech Pathology and Audiology, National Institute of Mental Health and Neurosciences (NIMHANS), a neuropsychiatry tertiary care hospital in Bangalore, Karnataka, India, from June 30, 2020, to December 23, 2023. The study was approved by the NIMHANS Ethics Committee (approval number: NIMH/DO/ (BS & NS DIV.)/2020-21). Informed consent was obtained from all the participants. 

Study population

Male and female patients aged 18-55 years, with a diagnosis of unilateral VS (right- or left-sided) on magnetic resonance imaging (MRI), were referred for pre-surgical audiological evaluation from the Department of Neurosurgery, NIMHANS.

Age- and gender-matched healthy individuals constituted the control group. Individuals with middle ear pathology, a history of neurological disease, or the use of vestibular suppressants were excluded from the study. Participation was voluntary, and all were right-handed. A priori sample size calculation was performed using GPower 3.1, and all consecutive patients presenting were included.

Test procedure

A trained member of the Gamma Knife Radiosurgery (GKRS) team used Leksell Gamma Plan (LGP) version 11.3, included with the Leksell Gamma Knife® Icon™ (Elekta AB, Stockholm, Sweden), to determine the tumour volume. 

The caloric and oculomotor tests were performed using VNG equipment (ICS Chartr 200; GN Store Nord A/S, Ballerup, Denmark). The participants were comfortably seated in a dark room, with the test goggles firmly strapped to their heads. After detecting the pupils and the region of interest, the individual's eye movements were calibrated in the horizontal and vertical planes. The distance between the light bar and the subject was kept at 3.8-4.2 feet. The following tasks were carried out. The oculomotor tests always preceded the caloric tests. 

Caloric Test

The participants were positioned supine with the head elevated to 30°. The process involved bi-thermal air irrigation of the external auditory canal at pre-set temperatures (24 °C for cool and 50 °C for warm) for 60 seconds per stimulation. The side of irrigation was randomly selected. Cool irrigation of both sides (one after the other) was done first and was followed by warm. Throughout the test, participants had their eyes open and counted backwards from 100 to maintain alertness. A five-minute rest period (eyes closed) was given between successive irrigations. Jongkee’s Formula [12] was used to calculate UWs and DP. The maximum SPV was analysed for each irrigation condition. Total SPV for each ear was computed as the sum of cool and warm SPVs and included in the analysis. 

*Oculomotor Tests* 

Saccade test: Participants were asked to follow the target on the light bar in the horizontal (10° and 15°) and vertical (10° and 15°) planes without moving their head. Each task was performed for at least 20 seconds. The average rightward and leftward (right and left eye) eye peak velocity, accuracy, and latency were considered for the analysis. 

Smooth pursuit test: Participants were asked to follow the target on the light bar in both the horizontal and vertical planes without moving their heads. The task was performed at 20 Hz, 30 Hz, 40 Hz, 50 Hz, 60 Hz, and 70 Hz, with a minimum of three cycles per frequency. In each frequency, the VOR gain for rightward and leftward movement (right and left eye) of the second cycle was considered for analysis. 

Optokinetic test: The participants were asked to look at the light bar (but not to stare or follow) as it crossed the field of vision (20°/second in the rightward and leftward directions) without moving their heads. This produces an optokinetic-induced nystagmus. A minimum of 20-second recordings were considered for each direction. The average SPV of the nystagmus between five seconds and 15 seconds (Total: 10 seconds) was considered for analysis.

Statistical analysis

Non-parametric tests were carried out as the data did not follow a normal distribution on the Shapiro-Wilk test. The patient group was divided into two groups: Rt-VS and Lt-VS. The Mann-Whitney U test was used to check if the tumor volume differed between the patient groups. The test metrics (oculomotor and caloric) were compared between the control and the patient groups using the Mann-Whitney U test. The level of significance considered for all the tests was <0.05. IBM SPSS Statistics for Windows, version 27 (IBM Corp., Armonk, New York, United States) was used for the statistical analysis.

## Results

Oculomotor and caloric tests were performed on patients with unilateral VS, and their performances were compared with those of age- and gender-matched healthy controls. There were no significant differences between the groups on age and gender, and between the patient groups on tumor volume (Table 1).

**Table 1:** Demographics of the control and the patient groups Koos: Koos classification of  Vestibular Schwannoma [13]

Characteristics		Control (n=120)	Right tumor group (n=60)				Left tumor group			
			Koos I (n=7)	Koos II (n=8)	Koos III (n=6)	Koos IV (n=39)	Koos I (n=8)	Koos II (n=12)	Koos III (n=3)	Koos IV (n=39)
Age	Mean	41.97	36.86	43.50	47.83	41.23	48.50	43.50	47.67	39.21
	SD	9.73	11.22	7.50	6.97	9.31	6.30	9.38	2.08	10.50
Sex	Male	64	4	3	3	19	7	7	2	22
	Female	56	3	5	3	20	1	5	1	17
Tumor Volume	Mean	-	0.34	1.27	2.52	17.34	0.27	0.95	2.36	13.68
	SD		0.14	0.47	0.37	13.49	0.12	0.46	0.30	8.54

Caloric test 

When compared to controls, the Rt-VS group revealed significant differences (p<0.05) for SPV of right ear cool and right warm stimulation, total right SPV, and UW. In the Lt-VS group, significant differences (p<0.05) were observed for left ear cool and warm stimulation, total SPV, right ear warm SPV stimulation, UW, and DP (Table 2).

**Table 2:** Comparison between control and patient groups on caloric test *significant (p=<0.05) Lt: left-sided; Rt: right-sided; VS: vestibular schwannoma; SPV: slow phase velocity

Test parameters	Control vs Rt-VS group		Control vs Lt-VS group	
	Z	p-value	Z	p-value
Right cool SPV	-6.494	<0.001*	-0.357	0.721
Left cool SPV	-0.030	0.976	-5.357	<0.001*
Right warm SPV	-6.070	<0.001*	-2.584	0.010*
Left warm SPV	-0.905	0.365	-5.829	<0.001*
Total right SPV	-6.638	<0.001*	-1.938	0.053
Total left SPV	-0.544	0.586	-5.980	<0.001*
Unilateral weakness	-7.418	<0.001*	-7.498	<0.001*
Directional preponderance	-1.514	0.130	-2.520	0.012*

Saccade test

Comparison between patients and controls revealed significant differences (p < 0.05) in both horizontal and vertical saccadic parameters in the Lt-VS and Rt-VS groups (Table 3). In the Rt-VS group, significant differences in the horizontal plane (10° and 15°) were observed for the right eye in rightward and leftward peak velocity, accuracy, and latency, and for the left eye in leftward peak velocity, accuracy, and latency, along with rightward latency. In the vertical plane, significant differences were noted for the right eye at 10° (upward peak velocity and latency; downward latency) and 15° (upward latency; downward accuracy and latency), and for the left eye at 10° (upward and downward latency) and 15° (upward latency; downward accuracy and latency).

**Table 3:** Comparison between control and patient groups on saccade test *significant (p=<0.05) Lt: left-sided; Rt: right-sided; VS: vestibular schwannoma

Test parameters	Control vs Rt-VS				Control vs Lt-VS			
	Right eye		Left eye		Right eye		Left eye	
	Z	p-value	Z	p-value	Z	p-value	Z	p-value
Horizontal 10 degrees								
Rightward peak velocity	-3.243	0.001*	-1.518	0.129	-.712	0.476	-1.869	0.062
Leftward peak velocity	-3.790	<0.001*	-3.079	0.002*	-1.763	0.078	-2.493	0.013*
Rightward accuracy	-2.626	0.009*	-0.764	0.445	-2.071	0.038*	-2.657	0.008*
Leftward accuracy	-3.629	<0.001*	-2.776	0.006*	-.462	0.644	-1.378	0.168
Rightward latency	-3.169	0.002*	-3.382	0.001*	-3.612	<0.001*	-4.642	<0.001*
Leftward latency	-3.995	<0.001*	-4.350	<0.001*	-4.442	<0.001*	-5.304	<0.001*
Horizontal 15 degrees								
Rightward peak velocity	-3.438	0.001*	-1.907	0.056	-1.191	0.234	-.101	0.919
Leftward peak velocity	-3.637	<0.001*	-3.121	0.002*	-0.935	0.350	-1.882	0.060
Rightward accuracy	-2.116	0.034*	-1.018	0.308	-1.089	0.276	-1.525	0.127
Leftward accuracy	-2.658	0.008*	-3.214	0.001*	-1.086	0.278	-2.320	0.020*
Rightward latency	-3.979	<0.001*	-4.779	<0.001*	-4.595	<0.001*	-5.683	<0.001*
Leftward latency	-3.431	0.001*	-4.756	<0.001*	-4.512	<0.001*	-5.458	<0.001*
Vertical 10 degrees								
Upward peak velocity	-2.457	0.014*	-0.110	0.912	-0.403	0.687	-0.317	0.751
Downward peak velocity	-1.466	0.143	-0.865	0.387	-1.074	0.283	-0.399	0.690
Upward accuracy	-1.551	0.121	-1.392	0.164	-2.599	0.009*	-1.928	0.054
Downward accuracy	-0.935	0.350	-1.097	0.273	-0.684	0.494	-0.816	0.415
Upward latency	-5.095	<0.001*	-5.341	<0.001*	-3.454	0.001*	-4.303	<0.001*
Downward latency	-5.358	<0.001*	-6.165	<0.001*	-4.597	<0.001*	-4.656	<0.001*
Vertical 15 degrees								
Upward peak velocity	-0.288	0.774	-1.183	0.237	-0.591	0.554	-0.407	0.684
Downward peak velocity	-0.137	0.891	-0.834	0.404	-0.855	0.393	-0.112	0.911
Upward accuracy	-1.319	0.187	-1.718	0.086	-2.991	0.003*	-2.057	0.040*
Downward accuracy	-3.622	<0.001*	-3.097	0.002*	-1.523	0.128	-1.764	0.078
Upward latency	-2.186	0.029*	-2.851	0.004*	-3.127	0.002*	-3.501	<0.001*
Downward latency	-4.984	<0.001*	-4.830	<0.001*	-5.077	<0.001*	-5.870	<0.001*

In the Lt-VS group, horizontal plane differences (10° and 15°) were observed for both right and left eye rightward and leftward latency. Additionally, left eye revealed significant difference for leftward peak velocity, rightward accuracy at 10°, and leftward accuracy at 15°, In the vertical plane, significant differences were found for the right eye at both 10° and 15° in upward accuracy and latency and downward latency, and for the left eye at 10° in upward and downward latency, and at 15° in upward accuracy and latency and downward latency

Smooth pursuit test

Patients (Rt-VS and Lt-VS) and controls revealed significant differences (p < 0.05) across all test frequencies (20, 30, 40, 50, 60, and 70 Hz) on horizontal smooth pursuit gain for both rightward and leftward eye movements in both eyes, except for the rightward 20 Hz in the Lt-VS group. For vertical smooth pursuit, the Rt-VS group showed significant differences (p < 0.005) in the right eye downward frequencies at 20, 30, 40, 50, and 70 Hz and upward frequencies at 20, 30, 40, 60 Hz. In the left eye, significant differences were observed for downward movements at 20, 40, 50, and 70 Hz and for upward movements across all frequencies (20-70 Hz). In contrast, the left tumour group did not show any significant differences in vertical smooth-pursuit gain for either eye during upward or downward movements (Table 4). 

**Table 4:** Comparison between control and patient groups on smooth pursuit test *significant (p=<0.05) Lt: left-sided; Rt: right-sided; VS: vestibular schwannoma

Test parameters	Control vs Rt-VS group				Control vs Lt-VS group			
	Right eye		Left eye		Right eye		Left eye	
	Z	p-value	Z	p-value	Z	p-value	Z	p-value
Horizontal								
Rightward 20 Hz	-6.054	<0.001*	-6.499	<0.001*	-1.790	0.074	-3.041	0.002*
Leftward 20 Hz	-4.133	<0.001*	-5.112	<0.001*	-3.391	0.001*	-3.857	<0.001*
Rightward 30 Hz	-4.382	<0.001*	-4.685	<0.001*	-4.377	<0.001*	-4.526	<0.001*
Leftward 30 Hz	-4.623	<0.001*	-5.178	<0.001*	-4.887	<0.001*	-5.385	<0.001*
Rightward 40 Hz	-6.157	<0.001*	-6.203	<0.001*	-4.085	<0.001*	-4.503	<0.001*
Leftward 40 Hz	-5.960	<0.001*	-5.959	<0.001*	-3.959	<0.001*	-4.251	<0.001*
Rightward 50 Hz	-6.943	<0.001*	-6.650	<0.001*	-3.227	0.001*	-3.060	0.002*
Leftward 50 Hz	-7.186	<0.001*	-7.071	<0.001*	-4.712	<0.001*	-4.453	<0.001*
Rightward 60 Hz	-6.293	<0.001*	-6.829	<0.001*	-4.215	<0.001*	-4.368	<0.001*
Leftward 60 Hz	-5.724	<0.001*	-5.849	<0.001*	-4.706	<0.001*	-4.614	<0.001*
Rightward 70 Hz	-5.724	<0.001*	-5.778	<0.001*	-4.926	<0.001*	-5.003	<0.001*
Leftward 70 Hz	-4.849	<0.001*	-4.038	<0.001*	-4.807	<0.001*	-4.891	<0.001*
Vertical								
Upward 20Hz	-2.166	0.030*	-3.116	0.002*	-1.332	0.183	-1.056	0.291
Downward 20Hz	-3.722	<0.001*	-4.705	<0.001*	-1.046	0.296	-0.099	0.921
Upward 30Hz	-2.272	0.023*	-2.196	0.028*	-0.858	0.391	-1.189	0.234
Downward 30Hz	-2.081	0.037*	-1.804	0.071	-0.835	0.404	-1.048	0.295
Upward 40Hz	-2.265	0.023*	-2.310	0.021*	-1.914	0.056	-1.621	0.105
Downward 40Hz	-2.284	0.022*	-2.692	0.007*	-0.303	0.762	-0.580	0.562
Upward 50Hz	-1.720	0.085	-2.237	0.025*	-0.641	0.521	-1.129	0.259
Downward 50Hz	-2.595	0.009*	-2.519	0.012*	-0.344	0.731	-1.474	0.141
Upward 60Hz	-2.734	0.006*	-2.464	0.014*	-0.126	0.900	-0.673	0.501
Downward 60Hz	-1.742	0.081	-1.746	0.081	-0.252	0.801	-0.653	0.513
Upward 70Hz	-2.183	0.029*	-2.261	0.024*	-0.470	0.639	-1.045	0.296
Downward 70Hz	-2.350	0.019*	-2.607	0.009*	-1.652	0.099	-1.586	0.113

Optokinetic test 

Average SPV for both rightward and leftward eye movements showed significant differences between patients (Rt-VS and Lt-VS groups) and controls (Table 5).

**Table 5:** Comparison between control and patient groups on optokinectic test *significant (p=<0.05) Lt: left-sided; Rt: right-sided; VS: vestibular schwannoma

Test parameters	Control vs Rt-VS group		Control vs Lt-VS group	
	Z	p-value	Z	p-value
Optokinectic_Rightward	-5.250	<0.001*	-3.221	0.001*
Optokinectic_Leftward	-4.956	<0.001*	-3.836	<0.001*

## Discussion

In this study, caloric and oculomotor test results were compared between 122 patients with unilateral VS (right and left) and 120 age- and gender-matched healthy controls. Patients showed clear abnormalities in both tests, indicating that both peripheral vestibular and central oculomotor involvement. Caloric tests induced VOR reflect mainly the integrity of the peripheral vestibular mechanism [8]. Through specific eye movements, the oculomotor test (saccades, smooth pursuit, and optokinetic) evaluates the central neural mechanism. Saccades, smooth pursuit, and optokinetic responses are indicators of central oculomotor function involving cortico-cerebellar-brainstem networks [2,14,15]. By employing both caloric and oculomotor tests, this study provides a comprehensive evaluation of peripheral and central mechanisms together. 

On caloric testing, patient groups (Rt-VS and Lt-VS) revealed unilateral vestibular hypofunction. The lesion-side gains (cool and warm stimulations) were significantly lower than those in the controls for both the Rt-VS and Lt-VS groups. On the non-lesion side, the Rt-VS group, as expected, did not significantly differ from the control group. At the same time, the Lt-VS group showed a significant difference in VOR gain for right-side (non-lesion) warm stimulation compared to the control group, which is less expected. However, this finding is to be seen in the context of UW. Both patient groups had significant UWs compared to the control group. UW reflects the overall interaural asymmetry across all stimulation conditions rather than isolated responses. UW findings in the current patient group assert the asymmetry and hence peripheral vestibular dysfunction. These findings are consistent with previous studies [16-18].

DP gives information on whether the right- or left-beating nystagmus is stronger. By DP, we postulate the directional bias of the vestibular system's output, and DP also serves as an objective marker for central vestibular compensation [8]. DP typically normalises with effective compensation; persistent elevation suggests incomplete or asymmetric central adaptation [6]. In the current study, unlike the Rt-VS group, the Lt-VS group did show a significant difference in DP compared to controls. This suggests that the side of the tumor (Right vs Left) has an impact on the central compensation. The persistent high DP in the Lt-VS group and the normal DP in the Rt-VS group may be due to right vestibular dominance. The vestibular system is right dominant, and hence the Rt-VS group had better central compensation [19].

The saccade test showed more pronounced abnormalities in eye movements toward the tumor side, characterized by increased latency, reduced peak velocity, and decreased accuracy in both the horizontal and vertical planes. Similar dysfunctions have been reported in patients with central vestibular and neuro-otological disorders [5]. Saccadic eye movements involve cortical, subcortical, cerebellar, and brainstem structures. An impaired cortical-subcortical processing time and delayed initiation of saccadic commands may cause the latency delay. The peak velocity and dysmetria indicate involvement of cerebellar and brainstem circuits [20,21]. In VS, the effect could be due to peripheral vestibular deafferentation and/or direct compression of central structures. Unlike small tumors, larger tumors, due to their size, extend into the cerebellopontine angle (CPA). It can compress the cerebellum and brainstem, leading to persistent oculomotor deficits [22]. In the current study, a major group of patients had large tumors with extension to CPA. Hence, the saccadic abnormalities may reflect less central compensation and more structural involvement.

Compared to the control group, both Rt-VS and Lt-VS had significantly reduced gain across all tested frequencies for horizontal smooth pursuit. This indicated impaired visual tracking ability and a compromised fronto-parieto-cerebellar circuit that integrates visual and vestibular information to maintain continuous tracking [14,23]. Vertical smooth pursuit deficits were more prominent in the Rt-VS group, suggesting the asymmetrical effect of VS based on the tumor side. The vertical gaze control pathway involves various brain stem nuclei, and it could be compromised [24]. Alterations in smooth pursuit gain have been observed in patients with vestibular deficits, suggesting sensory-motor recalibration [25]. The right vestibular pathway is reported to be the dominant side [26], and that could explain the higher deficits seen in the Rt-VS group. The cerebellum plays a critical role both in horizontal and vertical smooth pursuit [27]; the dysfunction observed in the current study could be due to the direct impact of the tumor on the cerebellum and its connecting vestibular pathway.

The SPV of the optokinetic nystagmus (OKN) was significantly reduced in both Rt-VS and Lt-VS groups. OKN reflects integration of cortical visual inputs with brainstem and cerebellar pathways, particularly involving the nucleus of the optic tract and vestibular nuclei [15]. In addition to a reduction in SPV, the OKN gain was significantly less in the patient groups. It implies an impaired velocity storage mechanism. Previous studies have reported abnormalities in optokinetic responses in patients with VS, supporting the presence of central involvement [28].

Study limitations

While this study provides comprehensive data from a large cohort, its cross-sectional design prevents longitudinal tracking of central compensation and functional recovery. Additionally, the clinical focus was primarily on tumor volume rather than the specific nerve of origin or precise anatomical compression of the brainstem, which may influence oculomotor outcomes. Finally, although VNG offers objective metrics, integrating subjective patient-reported quality-of-life measures would provide a more holistic view of the functional impact of VS.

## Conclusions

The present findings further confirm the involvement of both peripheral and central structures in unilateral VS. The caloric test results indicate substantial peripheral vestibular deafferentation on the lesion side. Central involvement can be construed by abnormal saccadic latency, velocity, and accuracy, along with reduced smooth pursuit gain and optokinetic slow-phase velocity. Overall, these VNG findings suggest that VS is beyond a peripheral disorder but could also affect central mechanisms responsible for maintaining stable gaze.

Caloric tests, along with the oculomotor tests, provide a broader view of peripheral, central, and compensatory mechanisms. This becomes particularly relevant in larger tumors, where such findings can help inform surgical planning, follow postoperative changes, and guide rehabilitation. Overall, a comprehensive VNG assessment adds meaningful clinical insight into the management of VS. In the present study, the observed pattern of deficits suggests that larger tumors may be associated with greater central involvement, affecting oculomotor pathways.
